# Using Q-methodology to understand the perspectives and practical experiences of dermatologists about treatment difficulties of cutaneous leishmaniasis

**DOI:** 10.1186/s12879-020-05365-0

**Published:** 2020-09-01

**Authors:** Dindar S. Qurtas, Nazar Pauls Shabila

**Affiliations:** 1 Department of Internal Medicine, College of Medicine, Hawler Medical University, Erbil, Kurdistan Iraq; 2 College of Health Sciences, Hawler Medical University, Erbil, Kurdistan Iraq

**Keywords:** Cutaneous leishmaniasis, Treatment, Viewpoints, Dermatologists

## Abstract

**Background:**

During the outbreak of cutaneous leishmaniasis in the Kurdistan Region of Iraq that started in 2015, the course of the disease and the treatment were not consistent with the available literature. Physicians, particularly dermatologists, faced challenges with treating the cutaneous leishmaniasis lesions with high rates of treatment failure and resistance to treatment. We used Q-methodology to understand the range and diversities of opinions and the practical experiences of dermatologists about the treatment difficulties of cutaneous leishmaniasis.

**Methods:**

This Q-methodology study was carried out in Erbil, Kurdistan Region of Iraq, and involved 37 dermatologists. A set of 40 statements related to different aspects of difficulties and uncertainties of treating cutaneous leishmaniasis was prepared. The dermatologists were requested to distribute the 40 statements into a scaled grid of nine piles from least agree to most agree. We applied by-person factor analysis using PQMethod 2.35 for the data analysis.

**Results:**

The analysis revealed two different viewpoints about the treatment of cutaneous leishmaniasis and a consensus viewpoint. The first viewpoint emphasized the use of sodium stibogluconate-based combination therapy, concerns with treatment failure, and lack of compliance with the treatment. The second viewpoint emphasized the lack of standard treatment and advances in the treatment of cutaneous leishmaniasis. There was a consensus between both groups of respondents about many aspects of the treatment of cutaneous leishmaniasis, including considering sodium stibogluconate the first drug of choice for cutaneous leishmaniasis treatment.

**Conclusions:**

This study revealed a diversity of viewpoints and uncertainties about the effectiveness of the available treatment modalities and treatment difficulties and failure. Interrupted supply and poor quality of the available drugs and lack of a standard and advanced treatment are the main problems facing the treatment of cutaneous leishmaniasis. More research is required to determine the best treatment modalities for the different types of cutaneous leishmaniasis. There is a need for the development of treatment guidelines specific to the Iraqi context with a particular focus on the treatment of the resistant and atypical cases of cutaneous leishmaniasis.

## Background

Cutaneous leishmaniasis (CL) is a vector born parasitic disease transmitted by a sandfly. The causative agent is the protozoa of *Leishmania* species [1]. The annual incidence of CL globally is about 1.5 million cases [2, 3]. Iraq and the neighboring countries are regarded as endemic areas for CL [4, 5]. The outbreak of this disease occurred in Erbil governorate in the Kurdistan Region of Iraq in 2015, which exerted a load on the health institutions to treat the cases. This outbreak was primarily related to massive population displacement, conflict in the surrounding area, and people’s movement from and to the endemic and affected areas [6].

Generally, the skin lesions of CL appear after an incubation period of about 3 months. The lesions usually appear at the sites of insect bit, which starts as a papule. Then papules increase in diameter to become nodules or plaques, which can ulcerate. According to the causative species of *Leishmania*, the lesions could be dry or wet; or multiple or limited in number [7]. Old World CL, mainly when the causative species is *Leishmania major*, one of the two species present in Iraq, is a mostly self-limiting skin disease in adults and can resolve within 6 months [8]. Based on a previous local study documenting Erbil 2015 outbreak, the disease could prolong more than 1 year even with the treatment [7, 9].

CL is diagnosed first by clinical suspicion then referred to the microscopy of skin scrap from the lesions, culturing in NNN medium and PCR technology. Isoenzyme technique is a gold standard for the differentiation of species, but unfortunately, this method is expensive and time-consuming. Another suggested modern and applicable molecular method of species detection in CL is polymerase chain reaction (PCR) [10–12]. Determination of *Leishmania* species is of considerable significance because the clinical presentations and treatment are highly reliable on the causative species. However, the diagnosis of CL in most developing countries is based on clinical presentations and microscopy smear because of a lack of investigation facilities [11, 13].

There is a wide range of local and systemic treatment options for CL [11]. Treatment modalities include local treatment and systematic treatment. Examples of local treatment include intralesional sodium stibogluconate (pentostam), intralesional metronidazole, paromomycine cream, and imiquimod cream. Examples of systemic treatment include sodium stibogluconate, other antimonies, miltefosine, amphotericin B, ketoconazole, and azithromycin [14]. Sodium stibogluconate is still the first treatment choice, despite having many toxic and side effects, especially when given systemically [15–17]. However, no treatment is found to be completely effective, and there are emerging resistant cases of CL. The CL treatment is species directed. In many countries where the tools for this investigation are not available, the treatment is empirically directed [11, 13].

CL is regarded as a neglected disease and a significant public health problem [18]. There are minimal controlled studies done to develop an effective treatment [11]. During the outbreak of CL in Erbil city, neither the course of the disease nor the treatment was consistent with the available literature [9]. Therefore, we used Q-methodology to understand the range and diversities of opinions and the practical experiences of dermatologists about the treatment difficulties of CL.

## Materials and methods

### Design

This study is based on Q-methodology, which involves the combination of quantitative and qualitative methods to study subjectivity and preferences scientifically and systematically. Q-methodology characterizes common views among groups of individuals [19, 20]. In this method, the study participants rank order a set of statements regarding a topic into a quasi-normal distribution according to their opinions from least agree to most agree (e.g., from − 4 or − 5 to + 4 or + 5). Then, factor analysis is carried out to the individual viewpoints (or rankings) [19, 21].

The procedure for analysis involves an inverted factor analysis. Correlation is carried out between persons to group the participants with similar viewpoints together. Thus, Q-factor analysis involves correlating across individuals to provide information on similarities and differences in opinion on a specific topic [19, 21, 22].

As the reporting guidelines for the main study types do not include specific guidelines for Q-methodology studies, we followed the standard guidelines described by Watts and Sterner [23] and van Exel and Graaf [19].

### Setting and participants

This study was carried out in Erbil, Kurdistan Region, Iraq. A sample of 37 physicians working in the field of dermatology in Erbil city was selected to participate in the study. These physicians included dermatology specialists, dermatology specialist trainees, and dermatology residents. The sample was purposively selected to include dermatologists with different degrees, professional levels, and years of experience.

### Statements

Initially, we asked 12 open-ended questions to a sample of 17 dermatologists about the main challenges and difficulties facing the treatment of CL in their practice (Additional file 1). There are around 70 dermatologists in Erbil city. The 17 dermatologists were selected from the new practicing classes with the first time exposure to CL and its management from the 2015 outbreak. We aimed to obtain their opinions about the recent CL epidemic from their clinical experience irrespective of the literature. These 17 dermatologists were not included in the final sample of 37 dermatologists who completed the Q-methodology study. All the answers from the open-ended questions were transcribed and summarized by the first author. This process resulted in having 59 statements related to different treatment modalities of CL (Additional file 2). Two authors independently reviewed the statements for any similarities. Any repeated statements were removed, closely similar statements were merged, and statements with polar opposite views were discarded. Any differences or no matching between the two authors were reconciled by consensus. We aimed to have statements representing CL’s different aspects of treatment modalities, focusing on the uncertainty of the best treatment modality and the treatment difficulties. Finally, we selected 40 statements that possibly described and adequately covered the topic of CL treatment modalities in the Iraqi Kurdistan Region.

**Fig. 1:**
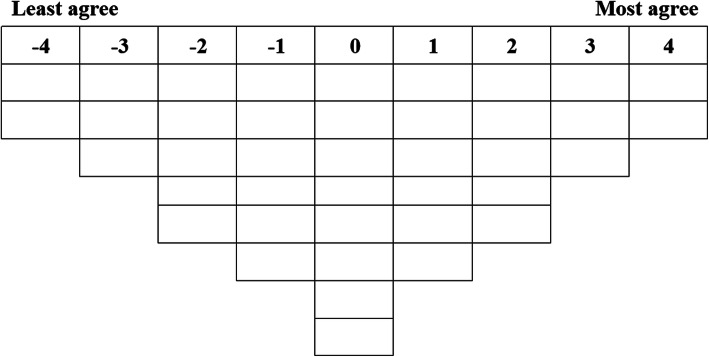
Scoring grid used for data collection

We numbered the 40 statements randomly and typed each statement on a small card. We developed a quasi-normal distribution Q-grid, including nine piles and 40 cells (Fig. 1), which was considered the data collection tool for this study.

### Data collection

We clarified the aim of the study to the respondents before providing consent to participate in the study. The participants were instructed to sort the statement cards into nine piles, ranging from − 4 (least agree) to + 4 (most agree), according to their viewpoints about various aspects and modalities of treating CL. Data were collected through one-to-one sessions. The participants thoroughly read each statement and ranked order the statements on the Q-grid cells. The study protocol was reviewed and approved by the Research Ethics Committee of Hawler Medical University.

### Data analysis

The PQMethod software program for Windows (release 2.35) was used for data management and analysis [24]. The 40 statements were entered into the program using STATES function (function 1). Then, the 37 Q-sorts made by the participants were entered into the program using the QENTER function (function 2). The apparent common viewpoints were obtained by centroid factor extraction (function 3) and varimax rotation (function 6). Centroid factor analysis involves defining centers of gravity embedded in a correlation matrix [25]. Varimax rotation involves placing factors to make the overall rotated solution account for the explained variance as much as possible. This step makes each Q-sort having a high factor loading on an individual factor alone, which helps in revealing the majority of viewpoints in the sample [23].

Any viewpoint that had at least two defining sorts and had an eigenvalue more than one were considered [26]. Defining Q-sorts are those that significantly load on one factor only. We selected a conservative significance level (*P* < 0.01) for the loading of the viewpoints. The Q-sorts with loading of ≥0.408 (2.58 × 1√number of statements) on a specific viewpoint were regarded as significant loading onto that viewpoint [27]. The Q-sorts with significant loading on more than an individual factor were considered confounded and were not labeled into any factor. The number of factor solutions was determined statistically. The analysis started with seven factors first. The analysis of seven, six, five, four, and three factors resulted in unsatisfactory solutions, i.e., the viewpoints produced had only a single respondent or no respondents that have loaded significantly on a particular viewpoint. Only the extraction of two factors provided more than a single respondent loading on each factor.

The final Q-analysis was carried out by the QANALYZE function of the program (function 7). From the correlation matrix, the weighted average scores of each viewpoint’s statements were obtained. The statements were initially sorted according to the scores. The statement scores were converted back into whole-number scores used in the initial sorting process (i.e., + 4, + 3, + 2, up to − 4). This step facilitated the comparison between the two factors.

The obtained viewpoints include the sorts of by the respondents who had responded principally in a similar way. Interpretation of the viewpoints was made subjectively by the examination of the characterizing and the distinguishing statements of each factor. A characterizing statement is a statement with a rank value at the extremes (i.e., most agree: + 4, + 3, and least agree: − 3, − 4). A distinguishing statement has a score on a viewpoint, which is significantly (*P* < 0.01) different from its score on another viewpoint. The distinguishing statements are highlighted with an asterisk (*) in the tables [19]. In the end, a conceptual interpretation was developed to label and describe the selected viewpoints.

## Results

Thirty seven physicians participated in the study with a mean ± SD age of 34.9 ± 7.2 years (range 27–63 years). Their mean ± SD years of experience was 6.5 ± 5.4 years (range 1–25 years). The details of the respondents’ demographic and professional characteristics are provided in Table 1.

**Table 1:** Demographic and professional characteristics of the respondents

Characteristic	No.	%
**Gender**		
Female	23	62.2
Male	14	37.8
**Age (years)**		
≤ 30	13	35.1
31–40	17	45.9
> 40	7	18.9
**Position**		
Dermatology resident	11	29.7
Dermatology specialist trainee	14	37.8
Dermatology specialist	12	32.4
**Years of experience**		
≤ 5	20	54.1
6–10	9	24.3
11–25	8	21.6

The analysis revealed two distinct viewpoints, which accounted for 48% of the variance. Twenty four respondents (64.9%) defined the two viewpoints. Four participants were confounded for loading significantly on both viewpoints, while nine participants did not load significantly on any viewpoint. The first viewpoint was labeled “sodium stibogluconate -based combination therapy with treatment failure concerns,” The second viewpoint was labeled “lack of standard or advanced treatment.” The sociodemographic characteristics and factor loading for each participant on two viewpoints are shown in Additional file 3.

### Viewpoint 1 – sodium stibogluconate-based combination therapy with treatment failure concerns

The first viewpoint accounted for 19% of the total variance. Sixteen respondents defined this viewpoint, including eight males and eight females, four specialists, eight specialist trainees, and four residents with a mean experience of 5.7 years. The distinguishing statements for the first viewpoint are shown in Table 2.

**Table 2:** Rank scores of distinguishing statements for views on the different modalities of cutaneous leishmaniasis treatment

#	Statement	View 1	View 2
1	Combination therapy of intralesional sodium stibogluconate and other CL therapy modalities such as cryotherapy, systemic antibiotics are superior to monotherapy with sodium stibogluconate.	+ 2*	−1
2	Combination therapy is superior to monotherapy for any case of CL	0*	−3
3	Effectiveness of combined intralesional sodium stibogluconate or cryotherapy with liquid nitrogen with antibiotics (e.g., Azithromycin or Doxycycline) is mainly due to overcoming secondary bacterial infection by antibiotics.	+ 2*	− 3
4	Problems of sodium stibogluconate are interrupted supply to health care providers and lack of original quality sometimes.	+ 3*	−1
5	Resistance to treatment in CL is due to low efficacy drugs.	+ 2*	+ 1
6	The use of herbal or homemade remedies by patients, which interfere with treatment, is very common.	+ 3*	−4
7	Patients noncompliance to the schedule of the treatment plan is the main obstacle for treating patients with CL	+ 2*	0
8	Fear of patients from treatment modalities and unawareness about the disease makes them not sticking to treatment schedules and instructions.	+ 1*	−1
9	If Zinc sulfate is given as monotherapy in large doses, it can heal CL patients.	−4*	−1
10	Rifampicin is a potent and effective anti- CL treatment.	−2*	0
11	Systematic antifungal drugs (e.g., ketoconazole or fluconazole) are effective in the treatment of CL.	−1*	0
12	Debridement and dressing of ulcerated lesions of CL have an important role in the treatment plan.	−3*	−2
13	For CL lesions of up to 4 in number, especially in hidden areas of the body, there is no need for any treatment, and only dressing and tying is enough.	−4*	−3
14	After decades of serious work and the presence of several options with newer compounds and combinations, there is still a little advance in the treatment of CL, which is not satisfactory for both doctors and patients.	−2*	+ 2
15	CL course extends more than 1–2 months despite treatment.	0*	+ 3
16	Resistance to treatment is the main complication of CL treatment.	−1*	+ 1
17	Cryotherapy with liquid nitrogen is the treatment of choice in children, especially for facial and ear lesions and dry lesions.	−1*	+ 3
18	The effect of cryotherapy with liquid nitrogen is unpredictable; some patients benefit from cryotherapy sessions while others come back with blistering and ulceration of lesions.	+ 1*	+ 2
19	Photodynamic therapy for CL lesions is advised for lesions located on cosmetically concerned areas such as the face.	−1*	+ 1
20	The intralesional sodium stibogluconate method is to inject it until the lesion turns white and is indurated.	+ 3*	+ 4
21	Systemic sodium stibogluconate is given when there is no response to cryotherapy with liquid nitrogen.	−2*	−4

*Distinguishing statement significant at < 0.01

*CL* cutaneous leishmaniasis

Respondents holding the first viewpoint believed in the superiority of combination therapy of intralesional sodium stibogluconate and other modalities to monotherapy with sodium stibogluconate (1; + 2 (statement 1; score + 2)) and the superiority of combination therapy to monotherapy in general (2; 0). They also emphasized that the effectiveness of combined intralesional sodium stibogluconate or cryotherapy with liquid nitrogen with an antibiotic is mainly due to overcoming secondary bacterial infection (3; + 2).

The respondents had concerns about the interrupted supply and low quality of sodium stibogluconate (4; + 3), development of resistance to treatment due to low efficacy of drugs (5; + 2), and the interference of frequent use of herbal of homemade remedies with treatment s (6; + 3). They also believed that patients’ noncompliance with the treatment plan is the main obstacle for treating CL (7; + 2), and the fear of patients of treatment modalities and unawareness about disease make them not to stick to treatment schedule and instructions (8; + 1).

In comparison to the second viewpoint, respondents holding the first viewpoint had less agreement about the ability of large doses of zinc sulfate as monotherapy to heal CL (9; − 4), the potency and effectiveness of rifampicin as anti-CL treatment (10; − 2), and the effectiveness of systematic antifungal drugs in treating CL (11; − 1). They also had less agreement with the role of debridement and dressing of ulcerated lesions in the treatment plan, (12; − 3), and dressing only for lesions up to 4 in number and on hidden areas of the body (13; − 4).

### Viewpoint 2 - lack of standard or advanced treatment

The second viewpoint accounted for 13% of the total variance. Eight participants defined the second viewpoint, including seven females and one male, three specialists, three specialist trainees, and two residents, with a mean experience of 3.2 years. The distinguishing statements for the second viewpoint are shown in Table 2.

In contrast with the first viewpoint, respondent holding the second viewpoint more strongly emphasized the presence of little advance in the treatment of CL even after decades of hard work and presence of several options with newer compounds and combinations (14; + 2), the extension of CL course than 1–2 months despite treatment (15; + 3), and the resistance to treatment as the main complication of CL treatment (16; + 1).

These respondents stressed on some technical aspects of treatment, including the use of cryotherapy with liquid nitrogen as the treatment of choice in case of children, especially for facial lesions and those which are located on the ears and dry lesions (17; + 3). They emphasized the unpredictable effect of cryotherapy with liquid nitrogen (18; + 2) and the use of photodynamic therapy for CL lesions when the lesions are located on cosmetically concerned areas like face (19; + 1).

The respondents believed that the intralesional sodium stibogluconate method involves injecting it until the lesion turns white and becomes indurated (20; + 4). They less agreed with giving systematic sodium stibogluconate when there is no response to cryotherapy with liquid nitrogen (21; − 4).

### Consensus statements

There was a general consensus between the two groups about many aspects of CL treatment, as shown in Table 3. Both groups very strongly agreed with considering sodium stibogluconate the first drug of choice for the treatment of CL’ (22; + 4, + 4 (statement 22, view 1: + 4, view 2: + 4) and lack of benefit from intralesional sodium stibogluconate injection in some patients even with many regular sessions (23; + 1, + 2). They believed in considering cryotherapy with liquid nitrogen as an alternative therapy to sodium stibogluconate for CL treatment (24; + 4, + 3) and avoiding cryotherapy with liquid nitrogen in wet and ulcerated lesions (25; + 2, + 2).

**Table 3:** Rank scores of consensus statements for views on the different modalities of cutaneous leishmaniasis treatment

#	Statement	View 1	View 2
22	Sodium stibogluconate is the first drug of choice for the treatment of CL^a^	+ 4	+ 4
23	Some patients do not benefit from intralesional sodium stibogluconate injection, even if many regular sessions are done^a^	+ 1	+ 2
24	Cryotherapy with liquid nitrogen is one of alternative therapy to sodium stibogluconate in the treatment of CL^a^	+ 4	+ 3
25	Cryotherapy with liquid nitrogen is better to be avoided in CL wet and ulcerated lesions^a^	+ 2	+ 2
26	Children suffering from CL are still a dilemma for available treatment modalities^a^	+ 1	+ 2
27	Majority of cases of CL are chronic since the morbidity last more up to and more than one year^a^	+ 1	+ 1
28	Most of the cases of CL get complete healing within 4–6 months of treatment, and rarely they extend more than one year^a^	+ 1	+ 1
29	Hypertonic saline intralesionally is of effective in the treatment of CL^a^	−3	−2
30	Relapses are very common after CL treatment^a^	−3	−2
31	Systemic sodium stibogluconate is given when there are big sized lesions^a^	−2	− 2
32	Systemic sodium stibogluconate is given when lesions are on cosmetically concerned site	−2	−2
33	Response to available CL treatment become evident and significant after two months of treatment^a^	0	0
34	Infrared therapy of CL lesions is a very good option of treatment either as monotherapy or in combination with other modalities^a^	0	+ 1
35	Zinc sulfate by mouth could be beneficial if given in combination with traditional therapy to strengthen immunity against CL^a^	0	0
36	Resistance to treatment in CL is due to the inappropriate way of treatment^a^	0	0
37	There is a controversy about when to decide systemic sodium stibogluconate for CL treatment. Still, it is not too clear for the physicians the indications for deciding the treatment^a^	−1	0
38	Intralesional metronidazole is effective anti CL treatment^a^	−1	0
39	Presence of resistant species of Leishmania, the emergence of new species or mutation in the previous species are the leading causes of failure of anti-leishmaniasis treatment especially sodium stibogluconate^a^	0	−1
40	Systemic sodium stibogluconate is given when there is no response to intralesional sodium stibogluconate^a^	0	−1

^a^ Consensus statement

*CL* cutaneous leishmaniasis

They considered children suffering from CL is still a dilemma for available treatment modalities (26; + 1, + 2), and the majority of cases of CL are chronic since the morbidity lasts more than 1 year (27; + 1, + 1). However, the thought that most CL cases get complete health within 4–6 months of treatment (28; + 1, + 1).

Both groups least agreed about the effectiveness of intralesional hypertonic saline in the treatment of CL (29; − 3, − 2), presence of frequent relapses after CL treatment (30; − 3, − 2), and giving systemic sodium stibogluconate for big sized lesions (31; − 2, − 2). They also least agreed with giving systemic sodium stibogluconate when lesions are on cosmetically concerned sites (32; − 2, − 2).

Both groups had neutral views about several aspects of CL treatment, including the need for 2 months for the treatment to become evident (33; 0, 0), the benefit of infrared therapy for the treatment of CL (34; 0, + 1), the benefit of zinc sulfate by mouth when given in combination with traditional therapy to strengthen immunity against CL (35; 0, 0), and development of resistance to CL treatment due to the inappropriate way of treatment (36; 0, 0).

Both groups had some neutral view or slight disagreement about the presence of controversy about the indications for systemic sodium stibogluconate for CL treatment (37; − 1, 0), and the effectiveness of intralesional metronidazole as anti-CL treatment (38; − 1, 0). The neutral view or slight disagreement was also about considering the presence of resistant species of *Leishmania*, and the emergence of new species or mutation as the leading cause of failure of anti-leishmaniasis treatment (39; 0, − 1), and giving systemic sodium stibogluconate when there is no response to intralesional sodium stibogluconate (40; 0, − 1).

## Discussion

This study revealed two distinct viewpoints among dermatologists regarding difficulties and uncertainties around the treatment of CL. The respondents holding the first viewpoint believed in using sodium stibogluconate-based combination therapy and have significant concerns with treatment failure and lack of compliance with the treatment. They were clearly against using homemade remedies or dressing and debridement alone.

The respondents having the first viewpoint believed in the superiority of combination therapy to monotherapy in general and, in particular, the superiority of combination therapy of intralesional sodium stibogluconate and other modalities to monotherapy with sodium stibogluconate. This is related to the synergistic effect of the combination of medications since no treatment is totally satisfactory for the treatment of CL. The combination therapy is recommended in endemic areas to avoid the development of resistant cases [28, 29]. They had concerns about the interrupted supply of sodium stibogluconate and its generics. Their concern is about the possibility of having reluctant cases to sodium stibogluconate and the need for a longer duration of the treatment course when the sodium stibogluconate is not provided regularly or substituted with generic preparations. The generic that has been used in our practice is sodium stibogluconate injection B.P., 30 mL vial, Albert David Ltd.

The respondents with the first viewpoint also had concerns about the interference of frequent use of herbal of homemade remedies with treatment. Such belief might be based on the fact that homemade remedies can sometimes cause severe side effects such as ulceration and dermatitis at the site of application. They had less agreement about the ability of large doses of zinc sulfate as monotherapy to healing CL. While the effectiveness of zinc sulfate is well documented [30, 31], the respondents’ setting experienced an atypical presentation of CL and failure to respond to available treatment modalities [9]. These respondents strongly disagreed with having dressing only for lesions up to four in number and on hidden areas of the body. However, the World Health Organization (WHO) manual for the treatment of CL in the Eastern Mediterranean region, which includes old world leishmaniasis species, mentions that lesions up to four in number, sizes up to 4 cm in diameter, and not on the face and fingers (situation I) could be left without any treatment [14]. At the same time, our dermatologists insist on treating even solitary lesions, which might be due to their frequent exposure to prolonged solitary lesions without resolution. This aspect is only true for the Old World leishmaniasis, as the disease can be localized and can have a tendency to spontaneous healing. However, it is generally accepted that the new world leishmaniasis, especially that caused by *Leishmania braziliensis*, should always be treated adequately to prevent disfiguring consequences in mucosal lesions [32].

The respondent holding the second viewpoint strongly emphasized the lack of standard treatment and advances in CL’s treatment. They were more concerned with the technical aspects of treatment. These respondents believed that there is a lack of advances in the treatment of CL. The pharmaceutical companies are less interested and encouraged to conduct well controlled studies and trials to develop novel treatments [11]. Therefore, the present treatment modalities, such as sodium stibogluconate, cryotherapy, zinc sulfate, photodynamic therapy, and metronidazole, are out of date [33].

The respondents having the second viewpoint thought that the use of cryotherapy with liquid nitrogen is the treatment of choice for children, especially for facial lesions and those located on the ears and dry lesions. As these areas of face and children are susceptible to pain, intralesional injection of sodium stibogluconate will be intolerable. On the contrary, cryotherapy is well tolerated and is effective in the treatment of CL [34]. They also believed that the effect of cryotherapy with liquid nitrogen is unpredictable because cryotherapy might give different cure rates (57.11 to 100%) [34–37]. Up to the best of our knowledge, there is no well-documented evidence about the role of cryotherapy in facial lesions.

The respondents with the second viewpoint believed that the intralesional sodium stibogluconate method is to inject it until the lesion turns white and is indurated. The most common documented procedure of quantifying the sodium stibogluconate dosage is induration and fading of the injected lesion [14]. They disagreed with giving systemic sodium stibogluconate when there is no response to cryotherapy with liquid nitrogen. Based on literature and from the dermatologists’ real-life experiences, sodium stibogluconate can cause toxicity and many side effects such as dizziness, headache, joint pain, hepatotoxicity, and cardiac arrest [38, 39]. These adverse effects may discourage dermatologists from deciding to shift to systemic sodium stibogluconate.

There was a general consensus between both groups of respondents about many aspects of the treatment of CL. Both groups considered sodium stibogluconate the first drug of choice for the treatment of CL. This finding is consistent with the up to date literature [15, 40]. The respondents thought that some patients do not benefit from intralesional sodium stibogluconate injection, even with many regular sessions. The presence of resistant cases of CL to sodium stibogluconate or variable degree of response is well-known [41, 42]. Thus, not every case treated with intralesional sodium stibogluconate or other therapies gets a benefit.

Both groups believed in considering cryotherapy with liquid nitrogen as an alternative to sodium stibogluconate in CL treatment. This perception of the doctors is consistent with available data, which confirms that cryotherapy, whether monotherapy or in combination with other modalities, is effective in CL treatment [34, 35]. The respondents least agreed about the effectiveness of intralesional hypertonic saline in the treatment of CL. This view might be attributed to experiencing many unresponsive cases in clinical practice, even with sodium stibogluconate. However, several research studies have suggested that hypertonic saline is effective in treating CL [43, 44].

The respondents did not agree with the presence of frequent relapses after CL treatment. They might be less acquainted with the long term course of the disease, as Erbil province was not endemic for CL before 2015 [6]. This attitude is not consistent with the literature about the leishmaniasis recidivans, which is the relapse of lesions after remission of the disease [45].

The emergence of new species or mutation of CL as a leading cause of anti-CL treatment failure was emphasized in this study. There are both *Leishmania tropica* and *Leishmania major* in Iraq. No microbiological studies have documented the occurrence of mutations in *Leishmania* species in Iraq. There is only one study from Erbil city that has reported unresponsiveness of some cases of CL to the standard treatment with sodium stibogluconate injections [9]. With the lack of documented local evidence about the mutation process or treatment resistance of CL in the Iraqi context, this idea might have been developed from a native clinical observation. The dermatologists might also have learned about such problems from other parts of the world.

This study has some potential limitations. Q-methodology is an exploratory tool, which helps in providing a useful insight into the existing views or opinions in society or a group of people. It also helps in characterizing each viewpoint. Q-methodology studies are not intended to generalize a finding or determine the proportion of individuals holding a particular opinion [46]. Therefore, our results were not presented within specific categories of gender, profession, years of experience, and other variables. However, these studies can provide an initial understanding of the sociodemographic and professional characteristics related to each opinion. Q-methodology can be considered a hypothesis-generating method that can help in designing larger surveys to assess the obtained viewpoints and their determinants [47]. Some aspects that were emphasized by the respondents are based entirely on clinical experiences in this particular setting rather than research-based evidence. An example of these aspects includes considering the emergence of new species or mutation as the leading cause of anti-leishmaniasis treatment failure. The conclusions and applicability of the findings of this study are related to the old world leishmaniasis. They should not be applied in other environments, such as the new world leishmaniasis.

## Conclusion

This study revealed a diversity of viewpoints and uncertainties about the effectiveness of the available treatment modalities and treatment difficulties and failure. Interrupted supply and poor quality of the available drugs and lack of a standard and advanced treatment are the main problems facing CL’s treatment. The distinguishing aspects between the two viewpoints on CL treatment difficulties and uncertainties and the consensus aspects were determined and described. Further research is required to determine the best treatment modalities for the different types of CL. There is a need for the development of treatment guidelines specific to the Iraqi context with a particular focus on the treatment of the resistant and atypical cases of CL.

## Supplementary information

**Additional file 1.** List of open-ended questions administered to 17 dermatologists to develop Q-statements.

**Additional file 2.** The set of the 59 initial statements extracted from the open-ended questions with 17 dermatologists.

**Additional file 3.** Participants’ characteristics and factor loading on the two factors.

## Data Availability

The datasets used for the current study are available from the corresponding author on a reasonable request.

## Abbreviation

CL: Cutaneous leishmaniasis
