# Supplementary material for: Using Q-methodology to understand the perspectives and practical experiences of dermatologists about treatment difficulties of cutaneous leishmaniasis
Source: BMC Infect Dis. 2020 Sep 1;20:645. doi: 10.1186/s12879-020-05365-0 (PMC7466828; doi:10.1186/s12879-020-05365-0)
Supplement: Supplementary file 1 — Additional file 1. List of open-ended questions administered to 17 dermatologists to develop Q-statements. [file 12879_2020_5365_MOESM1_ESM.docx]

**Additional file 1.** List of open-ended questions administered to 17 dermatologists to develop Q-statements

1. What are the treatment options available in your clinical practice for CL?
2. What is the first choice and most efficacious treatment option in your practice setting?
3. What is the role and the strength of cryotherapy with liquid nitrogen in the treatment of CL?
4. What is the role of dressing and debridement of ulcers of CL lesions in the treatment of CL?
5. When do you use systemic sodium stibogluconate in the treatment of CL?
6. Describe way you give intralesional sodium stibogluconate.
7. What types of complications of anti-CL treatment you have experienced in your practice?
8. What other treatment do you know for the treatment of CL apart from cryotherapy and sodium stibogluconate? Mention their efficacy in comparison with these two treatment.
9. What do you think about following WHO protocol for the treatment of CL in Iraq?

“When number of CL lesions is 4 or less, there is no need to treat leave the case to develop immunity for Leishmaniasis”

1. What is your opinion about chronicity of CL?
2. What are main problems of CL treatment?
3. What are the causes of resistance of CL to treatment?

CL: Cutaneous leishmaniasis
