# Supplementary material for: Using Q-methodology to understand the perspectives and practical experiences of dermatologists about treatment difficulties of cutaneous leishmaniasis
Source: BMC Infect Dis. 2020 Sep 1;20:645. doi: 10.1186/s12879-020-05365-0 (PMC7466828; doi:10.1186/s12879-020-05365-0)
Supplement: Supplementary file 2 — Additional file 2. The set of the 59 initial statements extracted from the open-ended questions with 17 dermatologists. [file 12879_2020_5365_MOESM2_ESM.docx]

**Additional file 2.** The set of 59 the initial statements extracted from the open-ended questions with 17 dermatologists

| **#** | **Statements** |
| --- | --- |
| 1 | Effectiveness of combined intralesional sodium stibogluconate or cryotherapy with liquid nitrogen with antibiotics (e.g., Azithromycin or Doxycycline) is mainly due to overcoming secondary bacterial infection by antibiotics† |
| 2 | Systematic antifungal drugs (e.g., ketoconazole or fluconazole) are effective in the treatment of cutaneous leishmaniasis† |
| 3 | Children suffering from CL are still a dilemma for available treatment modalities† |
| 4 | Combination therapy is superior to monotherapy for any case of cutaneous leishmaniasis† |
| 6 | Fear of patients from treatment modalities and unawareness about the disease makes them not to stick to treatment schedule and instructions† |
| 5 | Patients noncompliance to the schedule of the treatment plan is the main obstacle for treating patients with CL† |
| 7 | Pain during intralesional injection or cryotherapy is the main complication of CL treatment* |
| 8 | Relapses are very common after CL treatment† |
| 9 | Resistance to treatment is the main complication of CL treatment† |
| 10 | Systemic therapy complications are important complications of CL treatment* |
| 11 | Cryotherapy with liquid nitrogen is better to be avoided in CL wet and ulcerated lesions† |
| 12 | Cryotherapy with liquid nitrogen effect is unpredictable, some patients after sessions of cryotherapy get benefit while others come back with blistering and ulceration of lesions†+F18 |
| 13 | Cryotherapy with liquid nitrogen is one of alternative therapy to sodium stibogluconate in the treatment of CL † |
| 14 | Cryotherapy with liquid nitrogen is the treatment of choice in children, especially for facial and ear lesions and dry lesions† |
| 15 | Cryotherapy with liquid nitrogen should be given in sessions one week apart and freezing time of 5 seconds* |
| 16 | Local therapy could be done by curettage and excision of the CL lesion* |
| 17 | Debridement and dressing of ulcerated lesion of CL has an important role in the treatment plan† |
| 18 | CL course extends more than 1-2 months despite treatment† |
| 19 | Presence of resistant species of Leishmania, the emergence of new species or mutation in the previous species are the leading causes of failure of anti-leishmaniasis treatment especially sodium stibogluconate† |
| 20 | Keeping lesion of CL especially wet ulcerated lesion, just dry without dressing helps in quicker response.* |
| 21 | Response to available CL treatment become evident and significant after two months of treatment† |
| 22 | Majority of cases of CL are chronic since the morbidity last more up to and more than one year† |
| 23 | Most cases of cutaneous leishmaniasis get complete healing within 4-6 months of treatment, and they rarely extend more than one year† |
| 24 | Ulcerated CL lesions usually need more time to respond to treatment* |
| 25 | CL is endemic in Western parts of Erbil governorate* |
| 26 | Metronidazole intralesional can provide a 50% response rate in CL* |
| 27 | Intralesional metronidazole is effective anti-CL treatment† |
| 28 | Use of herbal or homemade remedies by patients which interfere with treatment is very common† |
| 29 | For lesions of CL up to 4 in number especially in hidden areas of the body, there is no need for any treatment and only dressing and tying is enough† |
| 30 | Not treating CL let the body to develop immunity against CL* |
| 35 | Combination therapy of intralesional sodium stibogluconate and other CL therapy modalities as cryotherapy, systemic antibiotics are superior to monotherapy with sodium stibogluconate† |
| 33 | The method of intralesional sodium stibogluconate is to inject it until the lesion turns white and is indurated† |
| 34 | In many cases despite regular standard intralesional sodium stibogluconate, the disease remains not improved after six months* |
| 31 | The method of intralesional sodium stibogluconate is to inject it until the lesion turns white and indurated. Overall it ought to be not more than 1 cc per lesion* |
| 32 | Some patients do not benefit from intralesional sodium stibogluconate injection, even if many regular sessions are done† |
| 36 | Monotherapy with sodium stibogluconate is usually ineffective in the treatment of CL* |
| 38 | Lack of original sodium stibogluconate is an important problem in the treatment of patients with CL* |
| 37 | Problems of sodium stibogluconate are interrupted supply to health care providers and lack of original quality sometimes† |
| 39 | The controversy is present on the opinions when deciding systemic sodium stibogluconate for CL treatment. Still, it is not too clear for the physician the indications upon which to decide treatment† |
| 40 | Lesions of CL more than 5 in number is one of the indications of systemic sodium stibogluconate* |
| 41 | Systemic sodium stibogluconate is given when lesions are on cosmetically concerned site† |
| 42 | Systemic sodium stibogluconate is given when there is no response to cryotherapy with liquid nitrogen† |
| 43 | Systemic sodium stibogluconate is given when the number of lesions is more than 10* |
| 44 | Systemic sodium stibogluconate is given when the patient is young or middle age* |
| 45 | Systemic sodium stibogluconate is given when there are big sized lesions† |
| 46 | Systemic sodium stibogluconate is given when there is no response to intralesional sodium stibogluconate† |
| 47 | Sodium stibogluconate is the first drug of choice for the treatment of CL† |
| 48 | Infrared therapy of CL lesions is a very good option of treatment either as monotherapy or in combination with other modalities† |
| 49 | Photodynamic therapy for CL lesions is advised when the lesions are located on cosmetically concerned areas like the face† |
| 50 | Radiofrequency, infra-red or Laser therapy are good options for the treatment of CL* |
| 51 | Resistance to treatment in CL is due to the inappropriate way of treatment† |
| 52 | Resistance to treatment in CL is due to incorrect diagnosis* |
| 53 | Resistance to treatment in CL is due to low efficacy drugs† |
| 54 | Resistance to treatment in CL is due to the use of homemade therapy by patient* |
| 55 | Rifampicin is a potent and effective anti- CL treatment† |
| 56 | Hypertonic saline intralesionally is of effective in the treatment of CL† |
| 57 | After decades of serious work and the presence of several options with newer compounds and combinations, there is still a little advance in the treatment of leishmaniasis and not satisfactory for both doctors and patients† |
| 58 | Zinc sulfate if it is given as monotherapy in large doses it can heal CL patients† |
| 59 | Zinc sulfate by mouth could be beneficial if given in combination with traditional therapy to strengthen immunity against CL† |

† Selected

* Discarded or combined with other statements
