# Supplementary material for: Using Q-methodology to understand the perspectives and practical experiences of dermatologists about treatment difficulties of cutaneous leishmaniasis
Source: BMC Infect Dis. 2020 Sep 1;20:645. doi: 10.1186/s12879-020-05365-0 (PMC7466828; doi:10.1186/s12879-020-05365-0)
Supplement: Supplementary file 3 — Additional file 3. Participants’ characteristics and factor loading on the two factors. [file 12879_2020_5365_MOESM3_ESM.docx]

**Additional file 3.** Participants’ characteristics and factor loading on the two factors

| **Participant No.** | **Age range (years)** | **Position** | **Experience (years)** | **Factor loading** | |
| --- | --- | --- | --- | --- | --- |
|  |  |  |  | **View 1** | **View 2** |
| 1 | 31-40 | Specialist trainee | 3 | **0.723^X^** | 0.055 |
| 2 | 21-30 | Specialist trainee | 5 | **0.712^X^** | 0.077 |
| 3 | 31-40 | Specialist | 11 | **0.630^X^** | -0.021 |
| 4 | 41-50 | Specialist | 17 | **0.628^X^** | 0.362 |
| 5 | 21-30 | Specialist trainee | 2 | **0.598^X^** | 0.300 |
| 6 | 31-40 | Specialist | 7.5 | **0.590^X^** | 0.385 |
| 7 | 31-40 | Specialist trainee | 5 | **0.570^X^** | 0.300 |
| 8 | 21-30 | Resident | 0.5 | **0.549^X^** | 0.085 |
| 9 | 31-40 | Specialist trainee | 9 | **0.549^X^** | -0.007 |
| 10 | 41-50 | Specialist | 10 | **0.548^X^** | 0.320 |
| 11 | 31-40 | Specialist trainee | 7 | **0.529^X^** | 0.270 |
| 12 | 21-30 | Resident | 3 | **0.523^X^** | 0.269 |
| 13 | 21-30 | Resident | 1.2 | **0.503^X^** | 0.176 |
| 14 | 31-40 | Specialist trainee | 4 | **0.498^X^** | 0.150 |
| 15 | 21-30 | Specialist trainee | 3.5 | **0.422^X^** | 0.102 |
| 16 | 31-40 | Resident | 2 | **0.409^X^** | 0.273 |
| 17 | 21-30 | Resident | 2 | 0.248 | **0.720^X^** |
| 18 | 21-30 | Resident | 1 | 0.042 | **0.563^X^** |
| 19 | 31-40 | Specialist trainee | 6 | 0.234 | **0.545^X^** |
| 20 | 31-40 | Specialist | 12 | 0.168 | **0.521^X^** |
| 21 | >50 | Specialist | 25 | 0.017 | **0.504^X^** |
| 22 | 31-40 | Specialist trainee | 7 | 0.267 | **0.477^X^** |
| 23 | 31-40 | Specialist trainee | 3 | -0.014 | 0.**453^X^** |
| 24 | 41-50 | Specialist | 15 | 0.083 | **0.446^X^** |
| 25 | 31-40 | Specialist trainee | 6 | **0.565** | **0.561** |
| 26 | 21-30 | Resident | 2 | **0.485** | **0.489** |
| 27 | 31-40 | Resident | 2 | **0.479** | **0.528** |
| 28 | 31-40 | Specialist | 10 | **0.431** | **0.469** |
| 29 | 21-30 | Specialist trainee | 4 | 0.401 | 0.271 |
| 30 | 21-30 | Resident | 2 | 0.401 | 0.178 |
| 31 | 31-40 | Resident | 2 | 0.385 | 0.298 |
| 32 | 41-50 | Specialist | 10 | 0.382 | 0.343 |
| 33 | 21-30 | Resident | 1 | 0.338 | 0.188 |
| 34 | 31-40 | Specialist | 11 | 0.225 | 0.267 |
| 35 | 41-50 | Specialist | 12 | 0.205 | 0.076 |
| 36 | 41-50 | Specialist | 13 | 0.149 | 0.406 |
| 37 | 21-30 | Specialist trainee | 2 | 0.083 | 0.016 |

Bold type indicates significant loadings. Significance at the 1% level is taken as a factor loading greater than (2.58 x 1√n), where n= the number of statements. In this case, significant loadings are those higher than 0.408.

^X^ indicates defining sorts.
